# Editorial: Hybrid Biomolecular Modeling

**DOI:** 10.3389/fmolb.2018.00098

**Published:** 2018-11-09

**Authors:** Slavica Jonic, Osamu Miyashita, Isabelle Callebaut

**Affiliations:** ^1^Sorbonne Université, UMR CNRS 7590, Muséum National d'Histoire Naturelle, IRD, Institut de Minéralogie, de Physique des Matériaux et de Cosmochimie, IMPMC, Paris, France; ^2^RIKEN Center for Computational Science, Kobe, Japan

**Keywords:** structure, dynamics, Conformational variability, hybrid models, biomolecules

Models of biomolecular structure and dynamics are often obtained by combining simulation or prediction approaches [e.g., comparative modeling, Molecular Dynamics (MD) simulations, Normal Mode Analysis (NMA), etc.] with experimental approaches [e.g., Nuclear Magnetic Resonance (NMR), X-ray crystallography, Small-Angle X-ray Scattering (SAXS), Electron Microscopy (EM), etc.] (Sali et al., 2015) (Figure 1). Such hybrid modeling extends the capabilities of experimental techniques, by enriching structural information and facilitating dynamics studies of biomolecules. This e-Book contains articles on methodological developments, applications, and challenges of hybrid biomolecular modeling that have been collected in the framework of the Frontiers Research Topic entitled “Hybrid Biomolecular Modeling.”

**Figure 1:**
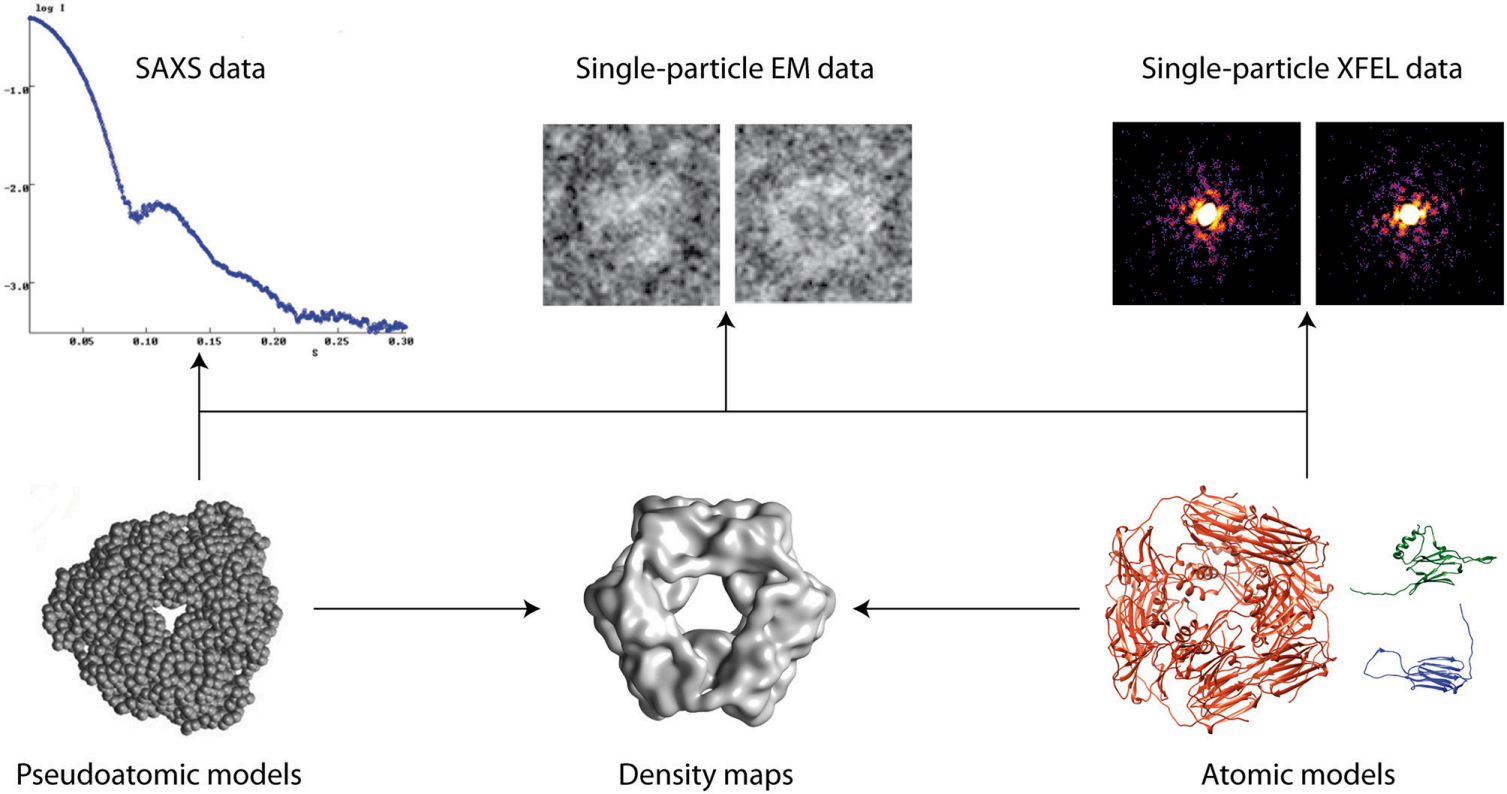
Hybrid modeling of biomolecular structures by fitting experimental data (arrows). Other data can also be used (e.g., NMR, cross-linking, FRET, etc.).

An example of hybrid modeling is fitting of structures of protein domains obtained by X-ray crystallography, NMR, or structure prediction into EM density maps of protein complexes (Kawabata, 2008; Birmanns et al., 2011; Tjioe et al., 2011; Yang et al., 2012). This allows obtaining high-resolution models of complexes when this cannot be achieved using a single experimental technique, as is often the case with large and flexible complexes (Cottevieille et al., 2008; Ciferri et al., 2012; Brown et al., 2014). This problem is addressed in the article by Habeck. The article is focused on a Bayesian inference approach to integrative biomolecular modeling by combining X-ray crystallography and cryo-EM data, but Habeck also discusses the computational challenges of this approach in a more general context of integrating other experimental data such as cross-linking/mass spectrometry and solid-state NMR data. The proposed approach is based on probabilistic models for cryo-EM maps and Markov chain Monte Carlo sampling of model structures from the posterior distribution.

Computational methods have been developed to predict the interactions between the protein subunits based on their shape complementary, electrostatic interactions, solvation energy, and statistical potential energy derived from the structural databases. This is known as molecular docking and one of its main challenges is the design of a reliable scoring function to assess the model quality. Inspired by the application of X-ray Free-Electron Lasers (XEFL) data in scoring models of conformational changes of complexes (Tokuhisa et al., 2016), Wang and Liu propose to use single-particle XFEL data for a more reliable scoring of models obtained by docking methods.

Computational approaches based on NMA or MD simulations have been developed to explore the conformational space of a model and identify the conformation (in this space) that best agrees with experimental data (Trabuco et al., 2008; Gorba and Tama, 2010; Jin et al., 2014). Devaurs et al. address this problem in the context of modeling based on experimental hydrogen/deuterium exchange (HDX) data. HDR data is often interpreted using an X-ray crystallography structure or a conformational ensemble obtained by MD simulations, though their correspondence with the HDR data is often not enough satisfactory. Devaurs et al. propose to select a single conformation that best fits the HDX data, from the conformational ensemble obtained with an extensive coarse-grained conformational sampling (of the given X-ray crystallography structure) that is biased with the information on the protein regions that produce the largest discrepancies with the HDX data.

Prischi and Pastore review an integrative structural modeling methodology that they have developed to determine the structure of weakly interacting molecular complexes. It combines NMR, SAXS, site directed mutagenesis, molecular docking, and MD simulations, and has been used by the authors and other groups to gain structural information on several iron-sulfur cluster (ISC) biogenesis complexes. The authors review these applications and discuss the advantages and limitations of this methodology as well as the future directions to improve it.

Woods et al. show a new application of an approach combining MD simulations, evolutionary sequence analysis, and Terahertz spectroscopy that they have developed to probe dynamics and allostery in rhodopsin. They show how the binding of the chlorophyll derivative, chlorin-e6 (Ce6) allosterically excites evolutionarily conserved communication pathways in rhodopsin that connect the ligand-binding site and the rest of the receptor.

Hsieh et al. present a NMA approach to analyze the dynamics of Dengue and Zika virus capsids based on their high-resolution cryo-EM models. They relate the differences identified in the dynamics of the E proteins in the two capsids to the differences observed in the two high-resolution models. They discuss the work that should be done in the future in order to fully characterize the dynamics of the two viruses.

Intrinsically disordered peptides and proteins present a challenge to experimental characterization of their functional conformations. Olson explores simulation techniques that could be used to build a computational framework for capturing conformational ensembles of such peptides and proteins. He explores temperature-based replica exchange methods for conformational ensemble sampling with implicit solvent models, as well as, explicit/implicit solvent hybrid replica exchange methods to capture the conformational ensemble of an intrinsically disordered peptide derived from the Ebola virus protein VP35. The author points out that intrinsically disordered peptides and proteins can be used as benchmarks to develop accurate methods for modeling conformational transitions.

The permeability of a cell membrane can be increased under the influence of an electric field of sufficient magnitude, which is known as membrane electroporation. Wriggers et al. address the problem of experimental and theoretical investigation of membrane electroporation. They extend, to the context of lipid bilayers and solvents, a statistical approach that they have originally developed for detecting allosteric signatures in MD simulations of well-structured proteins. This method is based on transforming time-domain information from MD trajectories into spatial heat maps that can be visualized on 3D molecular structures or in the form of interaction networks. The method is multiscale in the time domain and uses a mutual information approach for statistical bridging between the fast (local variables recorded by MD) and slow (global rate of change) time series. The mutual information method used with proteins was adapted to lipids and solvents by developing a new approach to probability density function estimation of random variables, which was described in a separate article (Kovacs et al.) in this e-Book.

We hope that this e-Book will be useful to experimentalists and method developers and that it will stimulate further use and development of hybrid biomolecular modeling methods. We thank all authors, co-authors, and reviewers for their contribution to this Research Topic and acknowledge the support from Frontiers Team members.

## Author contributions

All authors listed have made a substantial, direct and intellectual contribution to the work, and approved it for publication.

### Conflict of interest statement

The authors declare that the research was conducted in the absence of any commercial or financial relationships that could be construed as a potential conflict of interest.
